# CD45: a critical regulator in immune cells to predict severe and non-severe COVID-19 patients

**DOI:** 10.18632/aging.103941

**Published:** 2020-10-16

**Authors:** Mingming Jin, Nannan Shi, Meng Wang, Chunzi Shi, Shengjie Lu, Qing Chang, Shuang Sha, Yun Lin, Yingmin Chen, Hui Zhou, Kaiyi Liang, Xuyuan Huang, Yuxin Shi, Gang Huang

**Affiliations:** 1 Shanghai Key Laboratory of Molecular Imaging, Shanghai University of Medicine and Health Sciences, Shanghai 201318, China; 2 Department of Radiology, Shanghai Public Health Clinical Center, Fudan University, Shanghai 201508, China; 3 Key Laboratory of Molecular Imaging, Jiading Central Hospital, Shanghai University of Medicine and Health Sciences, Shanghai 201800, China; 4 Department of Infectious Disease, Shanghai Public Health Clinical Center, Fudan University, Shanghai 201508, China

**Keywords:** coronavirus disease 2019, COVID-19, severe acute respiratory syndrome coronavirus-2 (SARS-CoV-2), pneumonia, clinical characteristics, immunopathology

## Abstract

The ongoing outbreak of COVID-19 has been announced by the World Health Organization as a worldwide public health emergency. The aim of this study was to distinguish between severe and non-severe patients in early diagnosis. The results showed that the mortality of COVID-19 patients increased accompanied by age. Host factors CRP, IL-1β, hs-CRP, IL-8, and IL-6 levels in severe pneumonia patients were higher than in non-severe patients. CD3, CD8, and CD45 counts were decreased in COVID-19 patients. The results of this study suggest that the K-values of CD45 might be useful in distinguishing between severe and non-severe cases. The cut-off value for CD45 was -94.33. The K-values for CD45 in non-severe case were above the cut-off values, indicating a 100% prediction success rate for severe and non-severe cases following SARS-CoV-2 infection. The results confirmed that immune system dysfunction is a potential cause of mortality following COVID-19 infection, particularly for the elderly. CD45 deficiency dysfunction the naïve and memory T lymphocytes which may affects the long-term effectiveness of COVID-19 vaccines. K-values of CD45 might be useful in distinguishing between severe and non-severe cases in the early infection. May be CD45 could increase the diagnostic sensitivity.

## INTRODUCTION

The ongoing outbreak of severe acute respiratory syndrome coronavirus-2 (SARS-CoV-2), commonly referred to as coronavirus disease 2019 (COVID-19), has been announced by the World Health Organization (WHO) as a worldwide public health emergency [1]. More than 190 countries have reported COVID-19 infections and more than three million people are currently infected worldwide. Each country have more than 15,000 people died from COVID-19 including Italy, France, and Spain, and more than 50,000 people have died in the United States since the beginning of 2020. There are currently more than a million people infected in the United States. The continuous increase in the number of infections has led to a global economic depression [2].

COVID-19 pneumonia was first reported in Wuhan, Hubei Province, China, in December 2019, and was followed by an outbreak across Hubei Province and other areas in the country. In China, through the unremitting efforts of the entire population, COVID-19 infections were under control by early March 2020 [3]. Regardless of where it originated, COVID-19 is a global disaster. China's experience in treating COVID-19 patients may inform how other countries manage this outbreak. For highly suspected patients, the current recommended procedure is for individuals to self-isolate and wait for results from the coronavirus nucleic acid test as well as radiology examination. If an infection is confirmed, the individual should then enter the emergency ward for treatment [4].

According to WHO interim guidance on January 12, 2020, COVID-19 infections are categorized as severe, mild, or asymptomatic, as well as critical (acute respiratory distress syndrome [ARDS], sepsis, septic shock). Severe pneumonia cases are defined as patients with a respiratory rate > 30 breaths/min, severe respiratory distress, or SpO_2_ < 90% in room air. Poor prognostic factors include multilobular infiltration on chest imaging, lymphopenia, bacterial co-infection, smoking history, chronic medical conditions such as hypertension, and age > 60 years (MuLBSTA score) [5]. A number of studies have shown that infection immunopathology is important [6]. However, in terms of cellular immune function, how to distinguish between non-severe and severe cases is unclear. Although nucleic acid testing has been applied clinically to identify COVID-19 infection, distinguishing between non-severe and severe COVID-19 pneumonia cases directly affects patient survival and treatment options. Thus, the present study aimed to describe and compare the clinical characteristics of severe and non-severe cases of COVID-19 pneumonia from the Shanghai Public Health Clinical Center Affiliated to Fudan University. The results of this study will help reveal the immune changes caused by COVID-19 infection as well as distinguish between severe and non-severe cases.

## RESULTS

### Host factors associated with disease severity

**Figure 1:**
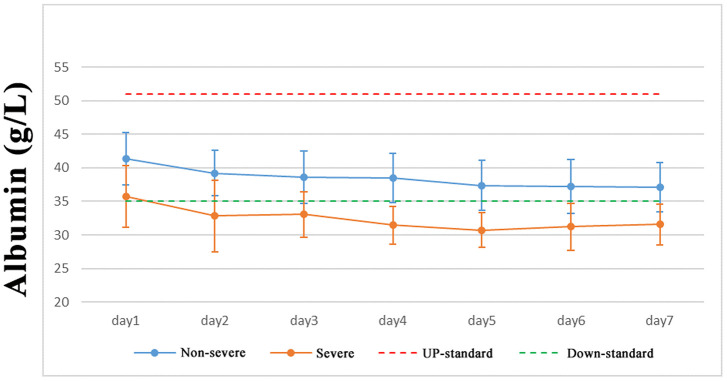
**Albumin expression in COVID-19 patients.**

As of March 9, 2020, we had 331 confirmed cases of COVID-19 in Shanghai, including 156 females and 175 males. The eldest was 88 years old and the youngest was 18 years old. The results showed that there was no difference in the rate of SARS-CoV-2 infection between the sexes. Some COVID-19 patients presented with different degrees of abnormality in liver function indexes. The serum levels of globulin, total bilirubin (TBIL), alkaline phosphatase, alanine transaminase, standard base deficit, potassium, and sodium in COVID-19 patients were not significantly different between non-severe and severe pneumonia patients. Albumin and total protein levels of most patients were within normal limits (Figure 1). In non-severe COVID-19 patients, total protein, albumin, and sodium levels were not significantly different across different age groups (< 50, 50–60, 60–70, and ≥ 70 years). However, total protein, albumin, and sodium levels were significantly lower in severe COVID-19 patients compared with non-severe COVID-19 patients (decrease of 26.9%, 42.3%, and 42.3%, respectively; Table 1), suggesting an abnormal liver function index in severe COVID-19 patients compared with non-severe COVID-19 patients.

**Table 1:** Clinical characteristics and laboratory results of liver function indexes for COVID-19 patients.

	**Non-severe**					**Severe**
**Age**	**<50**	**50~60**	**60~70**	**≥70**	**Overall**	**(N=26)**
**N**	**(N=159)**	**(N=58)**	**(N=65)**	**(N=23)**	**(N=305)**	**(N=26)**
**Sex**						
female	69(43.4%)	27(46.6%)	39(60.0%)	15(65%)	150(49.2%)	6 (23.1%)
male	90(56.6%)	31(53.4%)	26(40.0%)	8(35%)	155(50.8%)	20 (76.9%)
**Total protein (g/L)**						
high	11(6.9%)	0(0%)	2(3.1%)	0(0%)	13(4.3%)	0 (0%)
low	3(1.9%)	3(5.2%)	2(3.1%)	2(8.7%)	10(3.3%)	7 (26.9%)
normal	145(91.2%)	55(94.8%)	61(93.8%)	21(91.3%)	282(92.5%)	19 (73.1%)
**Albumin (g/L)**						
low	4(2.5%)	4(6.9%)	4(6.2%)	3(13%)	15(4.9%)	11 (42.3%)
normal	155(97.5%)	54(93.1%)	61(93.8%)	20(87%)	290(95.1%)	15 (57.7%)
**Globulin (g/L)**						
high	67(42.1%)	18(31.0%)	21(32.3%)	5(21.7%)	111(36.4%)	9 (34.6%)
normal	92(57.9%)	39(67.2%)	43 (66.2%)	18 (78.3%)	192 (63.0%)	0 (0%)
low	0(0%)	1 (1.7%)	1 (1.5%)	0 (0%)	2(0.7%)	17 (65.4%)
**TBIL (μmol/L)**						
high	6(3.8%)	4(6.9%)	3(4.6%)	0(0%)	13(4.3%)	4 (15.4%)
low	1(0.6%)	0(0%)	0(0%)	0(0%)	1(0.3%)	0 (0%)
normal	152(95.6%)	54(93.1%)	62(95.4%)	23(100%)	291(95.4%)	22 (84.6%)
**ALP (U/L)**						
high	2(1.3%)	0(0%)	4(6.2%)	1(4.5%)	7(2.3%)	1 (3.8%)
low	12(7.5%)	1(1.7%)	2(3.1%)	1(4.5%)	16(5.2%)	0 (0%)
normal	145(91.2%)	57(98.3%)	59(90.8%)	21(91%)	182(92.5%)	25 (96.2%)
**ALT (U/L)**						
high	30(18.9%)	13(22.4%)	6(9.2%)	2(8.7%)	51(16.7%)	4 (15.4%)
normal	129(81.1%)	45(77.6%)	59(90.8%)	21(91.3%)	254(83.3%)	22 (84.6%)
**SBC (mmol/L)**						
high	1(0.6%)	2(3.4%)	5(7.7%)	0(0%)	8(2.6%)	2(7.7%)
low	13(8.2%)	6(10.3%)	2(3.1%)	5(21.7%)	26(8.5%)	4(15.4%)
N	1(0.6%)	0(0%)	1(1.5%)	0(0%)	2(0.7%)	1(3.8%)
normal	144(90.6%)	50(86.2%)	57(87.7%)	18(78.3%)	269(88.2%)	19(73.1%)
**Potassium (mmol/L)**						
low	19(11.9%)	12(20.7%)	14(21.5%)	2(8.7%)	47(15.4%)	5(19.2%)
N	3(1.9%)	1(1.7%)	0(0%)	0(0%)	5(1.6%)	0(0%)
normal	137(86.2%)	45(77.6%)	51(78.5%)	21(91.3%)	253(83.0%)	21(80.8%)
**Sodium (mmol/L)**						
low	3(1.9%)	5(8.6%)	4(6.2%)	4(17.4%)	16(5.2%)	11(42.3%)
N	3(1.9%)	1(1.7%)	0(0%)	1(4.3%)	5(1.6%)	0(0%)
normal	153(96.2%)	52(89.7%)	61(93.8%)	18(78.3.0%)	284(93.1%)	15(57.7%)

TBIL, total bilirubin; ALP, alkaline phosphatase; ALT, alanine transaminase; SBD, standard base deficit.

Urine analysis of uric acid, blood urea nitrogen, urinary red blood cells, urine-pH, and urinary white blood cells showed that these factors were not significantly different between non-severe and severe pneumonia patients. However, urine protein and urine latent blood (BLD) were significantly increased in both non-severe and severe COVID-19 patients. Severe COVID-19 patients showed a greater increase in urine protein and BLD, suggesting kidney damage following SARS-CoV-2 infection (Table 2). Data analysis found that chlorine levels in non-severe patients were significantly increased. Chlorine, the main anion in the extracellular fluid of the human body, regulates the acid-base balance, osmotic pressure, and water distribution. Decreased chlorine levels indicate kidney damage. However, the kidney function indexes of non-severe COVID-19 patients across different age groups (< 50, 50–60, 60–70, ≥ 70 years) were not significantly different (Table 2).

**Table 2:** Urine test predicts kidney function for COVID-19 patients.

	**Non-severe**					**Severe**
**Age**	**<50**	**50~60**	**60~70**	**≥70**	**Overall**	**(N=26)**
**N**	**(N=159)**	**(N=58)**	**(N=65)**	**(N=23)**	**(N=305)**	**(N=26)**
**Uric acid (μmmol/L)**						
high	13(8.2%)	4(6.9%)	3(4.6%)	4(17.4%)	24(7.9%)	4 (15.4%)
low	2(1.3%)	3(5.2%)	0(0%)	1(4.3%)	6(2.0%)	4 (15.4%)
normal	144(90.6%)	51(87.9%)	62(95.4%)	18(78.3%)	275(90.2%)	18 (69.2%)
**BUN (mmol/L)**						
high	4(2.5%)	3(5.2%)	9(13.8%)	4(17.4%)	20(6.6%)	7 (26.9%)
low	1(0.6%)	0(0%)	1(1.5%)	0(0%)	2(0.7%)	0 (0%)
normal	154(96.9%)	55(94.8%)	55(84.6%)	19(82.6%)	283(92.8%)	19 (73.1%)
**Urine protein**						
high	32(20.1%)	18(31.0%)	19(29.2%)	6(26%)	75(24.6%)	16 (61.5%)
N	35(22.0%)	10(17.2%)	11(16.9%)	3(13%)	59(19.3%)	3 (11.5%)
normal	92(57.9%)	30(51.7%)	35(53.8%)	14(61%)	171(56.1%)	7 (26.9%)
**URBC (cell/μl)**						
high	18(11.3%)	6(10.3%)	6(9.2%)	3(13%)	33(10.8%)	7 (26.9%)
N	36(22.6%)	10(17.2%)	12(18.5%)	3(13%)	61(20.0%)	3 (11.5%)
normal	105(66.0%)	42(72.4%)	47(72.3%)	15(74%)	211(69.2%)	16 (61.5%)
**BLD**						
high	26(16.4%)	15(25.9%)	12(18.5%)	5(21.7%)	58(19.0%)	11 (42.3%)
N	35(22.0%)	10(17.2%)	11(16.9%)	3(13.1%)	59(19.3%)	3 (11.5%)
normal	98(61.6%)	33(56.9%)	42(64.6%)	15(65.2%)	188(61.6%)	12 (46.2%)
**U-pH**						
N	35(22.0%)	10(17.2%)	11(16.9%)	3(13%)	59(19.3%)	2 (7.7%)
normal	124(78.0%)	48(82.8%)	52(80.0%)	20(87%)	244(80.0%)	3 (11.5%)
high	0(0%)	0(0%)	2(3.1%)	0(0%)	2(0.7%)	21 (80.8%)
**UWBC (cell/μl)**						
N	36(22.6%)	10(17.2%)	11(16.9%)	3(13%)	60(19.7%)	3 (11.5%)
normal	123(77.4%)	48(82.8%)	54(83.1%)	20(87%)	245(80.3%)	23 (88.5%)
**Chlorin (mmol/L)**						
high	58(36.5%)	21(36.2%)	26(40.0%)	9(39.1%)	114(37.4%)	4(15.4%)
N	3(1.9%)	1(1.7%)	0(0%)	1(4.3%)	5(1.6%)	0(0%)
normal	98(61.6%)	36(62.1%)	39(60.0%)	13(56.5%)	186(61.0%)	22(84.6%)

N, the data is incomplete; BUN, serum urea nitrogen; URBC, urinary red blood cell; BLD, urine latent blood; U-pH, urine-pH; UWBC, urine white blood cell.

**Figure 2:**
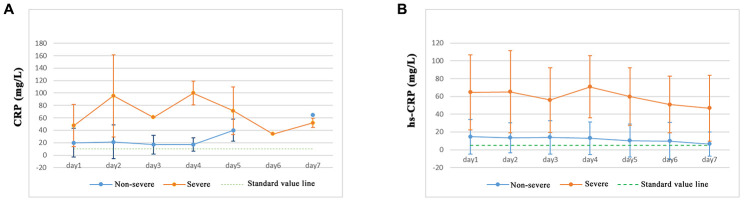
**Expression of CRP and hs-CRP in COVID-19 patients.**

Serum inflammatory factor levels of IL-2, IL-1β, IL-4, IL-6, IL-5, IL-10, IL-8, IL-12P70, IL-17, IFN-α, TNF-α, IFN-γ, and endotoxin in COVID-19 patients showed no significant differences between non-severe and severe pneumonia patients. However, IL-6, IL-1β, and IL-8 expression levels in severe pneumonia patients were increased, and the levels of IL-6 showed the greatest increase. The levels of C-reactive protein (CRP) and high-sensitivity C-reactive protein (hs-CRP) were significantly increased in both non-severe and severe pneumonia patients. A higher proportion of the severe pneumonia patient population had increased CRP and hs-CRP levels (Table 3). Severe pneumonia patients showed higher levels of CRP (Figure 2A) and hs-CRP (Figure 2B) in the first 7 days of analysis, suggesting that SARS-CoV-2 has bacterial infection characteristics. Serum inflammatory factor levels of IL-2, IL-1β, IL-5, IL-4, IL-6, IL-8, IL-10, IL-17, IL-12P70, IFN-α, TNF-α, IFN-γ, endotoxin, CRP, and hs-CRP of non-severe COVID-19 patients across different age groups (< 50, 50–60, 60–70, and ≥ 70 years) were not significantly different (Table 3).

**Table 3:** Inflammatory factors detection for COVID-19 patients.

	**Non-severe**					**Severe**
**Age**	**<50**	**50~60**	**60~70**	**≥70**	**Overall**	**(N=26)**
**N**	**(N=159)**	**(N=58)**	**(N=65)**	**(N=23)**	**(N=305)**	**(N=26)**
**CRP (mg/L)**						
high	65(40.9%)	40(69.0%)	36(55.4%)	15(65.2%)	156(51.1%)	26(100%)
N.A	3(1.9%)	1(1.7%)	1(1.5%)	0(0%)	5(1.6%)	0(0%)
normal	91(57.2%)	17(29.3%)	28(43.1%)	8(34.8%)	144(47.2%)	0 (0%)
**hs-CRP (mg/L)**						
high	76(47.8%)	39(67.2%)	42(64.6%)	18(78.3%)	175(57.4%)	24(92.3%)
N.A	3(1.9%)	2(3.4%)	1(1.5%)	1(4.3%)	7(2.3%)	2(7.7%)
normal	80(50.3%)	17(29.3%)	22(33.8%)	4(17.4%)	123(40.3%)	0(0%)
**IL-1β (pg/ml)**						
high	23(14.5%)	7(12.1%)	7(10.8%)	1(4.3%)	38(12.5%)	8(30.8%)
N	99(62.3%)	31(53.4%)	36(55.4%)	12(52.2%)	178(58.4%)	1(3.8%)
normal	37(23.3%)	20(34.5%)	22(33.8%)	10(43.5%)	89(29.2%)	17(65.4%)
**IL-2 (pg/ml)**						
high	1(0.6%)	0(0%)	0(0%)	0(0%)	1(0.3%)	3(11.5%)
N	61(38.4%)	15(25.9%)	17(26.2%)	6(26.1%)	99(32.5%)	1(3.8%)
normal	97(61.0%)	43(74.1%)	48(73.8%)	17(73.9%)	205(67.2%)	22(84.6%)
**IL-4 (pg/ml)**						
N	61(38.4%)	15(25.9%)	17(26.2%)	6(26.1%)	99(32.5%)	1(3.8%)
normal	98(61.6%)	43(74.1%)	48(73.8%)	17(73.9%)	206(67.5%)	25(96.2%)
**IL-5 (pg/ml)**						
N	99(62.3%)	31(53.4%)	36(55.4%)	12(52.2%)	178(58.4%)	1(3.8%)
normal	60(37.7%)	27(46.6%)	29(44.6%)	11(47.8%)	127(41.6%)	21(80.8%)
high	0(0%)	0(0%)	0(0%)	0(0%)	0(0%)	4(15.4%)
**IL-6 (pg/ml)**						
high	12(7.5%)	12(20.7%)	12(18.5%)	4(17.4%)	40(13.1%)	16(61.5%)
N	61(38.4%)	15(25.9%)	17(26.2%)	6(26.1%)	99(32.5%)	1(3.8%)
normal	86(54.1%)	31(53.4%)	36(55.4%)	13(56.5%)	166(54.4%)	9(34.6%)
**IL-8 (pg/ml)**						
high	3(1.9%)	0(0%)	1(1.5%)	0(0%)	4(1.3%)	9(34.6%)
N	99(62.3%)	31(53.4%)	36(55.4%)	12(52.2%)	178(58.4%)	1(3.8%)
normal	57(35.8%)	27(46.6%)	28(43.1%)	11(47.8%)	123(40.3%)	16(61.5%)
**IL-10 (pg/ml)**						
N	61(38.4%)	15(25.9%)	17(26.2%)	6(26.1%)	99(32.5%)	3(11.5%)
normal	98(61.6%)	43(74.1%)	46(70.8%)	17(73.9%)	204(66.9%)	1(3.8%)
high	0(0%)	0(0%)	2(3.1%)	0(0%)	2(0.7%)	22(84.6%)
**IL-12P70 (pg/ml)**						
high	2(1.3%)	1(1.7%)	0(0%)	0(0%)	3(1.0%)	1(3.8%)
N	99(62.3%)	31(53.4%)	36(55.4%)	12(52.2%)	178(58.4%)	1(3.8%)
normal	58(36.5%)	26(44.8%)	29(44.6%)	11(47.8%)	124(40.7%)	24(92.3%)
**IL-17 (pg/ml)**						
N	61(38.4%)	15(25.9%)	17(26.2%)	6(26.1%)	99(32.5%)	1(3.8%)
normal	98(61.6%)	43(74.1%)	48(73.8%)	17(73.9%)	206(67.5%)	24(92.3%)
high	0(0%)	0(0%)	0(0%)	0(0%)	0(0%)	1(3.8%)
**TNF-α (pg/ml)**						
high	2(1.3%)	0(0%)	0(0%)	0(0%)	2(0.7%)	0(0%)
N	61(38.4%)	15(25.9%)	18(27.7%)	6(26.1%)	100(32.8%)	1(3.8%)
normal	96(60.4%)	43(74.1%)	47(72.3%)	17(73.9%)	203(66.6%)	25(96.2%)
**IFN-α (pg/ml)**						
N	99(62.3%)	31(53.4%)	37(56.9%)	12(52.2%)	179(58.7%)	3(11.5%)
normal	60(37.7%)	27(46.6%)	28(43.1%)	11(47.8%)	126(41.3%)	23(88.5%)
**IFN-γ (pg/ml)**						
high	1(0.6%)	1(1.7%)	1(1.5%)	0(0%)	3(1.0%)	0(0%)
N	62(39.0%)	15(25.9%)	18(27.7%)	6(26.1%)	101(33.1%)	1(3.8%)
normal	96(60.4%)	42(72.4%)	46(70.8%)	17(73.9%)	201(65.9%)	25(96.2%)
**Endotoxin (pg/ml)**						
high	51(32.1%)	15(25.9%)	19(29.2%)	9(39.1%)	94(30.8%)	3(11.5%)
N	20(12.6%)	8(13.8%)	6(9.2%)	5(21.8%)	39(12.8%)	6(23.1%)
normal	88(55.3%)	35(60.3%)	40(61.5%)	9(39.1%)	172(56.4%)	17(65.4%)

CRP, C-reactive protein; hs-CRP, high-sensitivity C-reactive protein; IL-1β, interleukin-1β; TNF-α, tumor necrosis factor α; IFN-α, interferon-α; IFN-γ, interferon-γ.

### Cell subsets and immunoglobulin detection in COVID-19 patients

**Figure 3:**
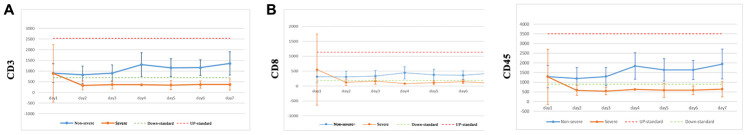
**CD3, CD8, and CD45 counts in COVID-19 patients.**

Routine blood tests showed that the following characteristics were significantly different between non-severe and severe pneumonia patients: red blood cells; hemoglobin; white blood cells (WBCs); basophil count; basophil percentage; monocyte and neutrophil count; CD4, CD8, CD4/CD8, CD9, and CD16/CD56 count/percentage; immunoglobulin A (IgA); and IgM. Eosinophil count/percentage, lymphocyte count/percentage, CD3 count/percentage, monocyte percentage, and CD45 count were decreased after SARS-CoV-2 infection. Severe pneumonia patients had lower CD3 (Figure 3A), monocyte, CD8 (Figure 3B), and CD45 (Figure 3C) counts in the first 7 days of analysis compared with non-severe patients. Different age group analysis found that lymphocyte, CD3, CD8, and CD45 counts were decreased in older SARS-CoV-2 patients, suggesting that immune function deteriorates with increasing age. The neutrophil percentage was also increased following SARS-CoV-2 infection, indicating that inflammatory infections are more severe (Table 4).

**Table 4:** Cell subsets and immunoglobulin detection for COVID-19 patients.

	**Non-severe**					**Severe**
**Age**	**<50**	**50~60**	**60~70**	**≥70**	**Overall**	**(N=26)**
**N**	**(N=159)**	**(N=58)**	**(N=65)**	**(N=23)**	**(N=305)**	**(N=26)**
**Hemoglobin (g/L)**						
high	46(28.9%)	13(22.4%)	1(1.5%)	1(0%)	61(20.0%)	5(19.2%)
low	6(3.8%)	4(6.9%)	7(10.8%)	3(0%)	20(6.6%)	3(11.5%)
normal	107(67.3%)	41(70.7%)	57(87.7%)	19(100%)	224(73.4%)	18(69.2%)
**RBC (×10^12^/L)**						
high	42(26.4%)	4(6.9%)	1(1.5%)	1(0%)	48(15.7%)	5(19.2%)
low	5(3.1%)	6(10.3%)	11(16.9%)	7(50.0%)	29(9.5%)	4(15.4%)
normal	112(70.4%)	48(82.8%)	53(81.5%)	15(50.0%)	228(74.8%)	17(65.4%)
**WBC (×10^9^/L)**						
high	4(2.5%)	0(0%)	1(1.5%)	0(0%)	5(1.6%)	5(19.2%)
low	37(23.3%)	14(24.1%)	16(24.6%)	9(0%)	76(24.9%)	7(26.9%)
normal	118(74.2%)	44(75.9%)	48(73.8%)	14(100%)	224(73.4%)	14(53.8%)
**Basophil (×10^9^/L)**						
normal	159(100%)	58(100%)	65(100%)	23(100%)	305(100%)	25(96.2%)
high	0(0%)	0(0%)	0(0%)	0(0%)	0(0%)	1(3.8%)
**Basophil percentage (%)**						
normal	159(100%)	58(100%)	65(100%)	23(100%)	305(100%)	25(96.2%)
high	0(0%)	0(0%)	0(0%)	0(0%)	0(0%)	1(3.8%)
**Eosinophil (×10^9^/L)**						
low	90(56.6%)	37(63.8%)	46(70.8%)	19(50%)	192(63.0%)	23(88.5%)
normal	69(43.4%)	21(36.2%)	19(29.2%)	4(50%)	113(37.0%)	3(11.5%)
**Eosinophil percentage (%)**						
low	103(64.8%)	41(70.7%)	53(81.5%)	19(50.0%)	216(70.8%)	23(88.5%)
normal	56(35.2%)	17(29.3%)	12(18.5%)	4(50.0%)	89(29.2%)	3(11.5%)
**Lymphocyte (×10^9^/L)**						
high	1(0.6%)	0(0%)	0(0%)	0(0%)	1(0.3%)	1(3.8%)
low	27(17.0%)	13(22.4%)	18(27.7%)	7(50.0%)	65(21.3%)	15(57.7%)
normal	131(82.4%)	45(77.6%)	47(72.3%)	16(50.0%)	239(78.4%)	10(38.5%)
**Lymphocyte percentage (%)**						
high	16(10.1%)	2(3.4%)	2(3.1%)	0(0%)	20(6.6%)	1(3.8%)
low	36(22.6%)	18(31.0%)	24(36.9%)	9(50%)	87(28.5%)	19(73.1%)
normal	107(67.3%)	38(65.5%)	39(60.0%)	14(50%)	198(64.9%)	6(23.1%)
**Monocyte (×10^9^/L)**						
high	8(5.0%)	4(6.9%)	3(4.6%)	1(0%)	16(5.2%)	4(15.4%)
normal	151(95.0%)	54(93.1%)	62(95.4%)	22(100%)	289(94.8%)	22(84.6%)
**Monocyte percentage (%)**						
high	90(56.6%)	37(63.8%)	41(63.1%)	17(100%)	185(60.7%)	8(30.8%)
low	2(1.3%)	0(0%)	0(0%)	0(0%)	2(0.7%)	4(15.4%)
normal	67(42.1%)	21(36.2%)	24(36.9%)	6(0%)	118(38.7%)	14(53.8%)
**Neutrophil (×10^9^/L)**						
high	3(1.9%)	0(0%)	1(1.5%)	0(0%)	4(1.3%)	4(15.4%)
low	50(31.4%)	15(25.9%)	17(26.2%)	7(0%)	89(29.2%)	5(19.2%)
normal	106(66.7%)	43(74.1%)	47(72.3%)	16(100%)	212(69.5%)	17(65.4%)
**Neutrophil percentage (%)**						
high	38(23.9%)	21(36.2%)	20(30.8%)	11(50.0%)	90(29.5%)	19(73.1%)
low	22(13.8%)	6(10.3%)	2(3.1%)	2(0%)	32(10.5%)	1(3.8%)
normal	99(62.3%)	31(53.4%)	43(66.2%)	10(50.0%)	183(60.0%)	6(23.1%)
**CD45 count (cell/μl)**						
high	1(0.6%)	1(1.7%)	1(1.5%)	0(0%)	3(1.0%)	0(0%)
low	18(11.3%)	11(19.0%)	13(20.0%)	7(50.0%)	49(16.1%)	15(57.7%)
N	2(1.3%)	0(0%)	0(0%)	0(0%)	2(0.7%)	0(0%)
normal	138(86.8%)	46(79.3%)	51(78.5%)	15(50.0%)	251(82.3%)	11(42.3%)
**CD3 count (cell/μl)**						
high	3(1.9%)	1(1.7%)	1(1.5%)	0(0%)	5(1.6%)	0(0%)
low	22(13.8%)	16(27.6%)	19(29.2%)	10(50.0%)	68(22.3%)	18(69.2%)
N	2(1.3%)	0(0%)	0(0%)	0(0%)	2(0.7%)	0(0%)
normal	132(83.0%)	41(70.7%)	45(69.2%)	11(50.0%)	230(75.4%)	8(30.8%)
**CD3 percentage (%)**						
high	6(3.8%)	2(3.4%)	2(3.1%)	0(0%)	10(3.3%)	0(0%)
low	3(1.9%)	2(3.4%)	3(4.6%)	1(0%)	9(3.0%)	6(23.1%)
N	2(1.3%)	0(0%)	0(0%)	0(0%)	2(0.7%)	0(0%)
normal	148(93.1%)	54(93.1%)	60(92.3%)	20(100%)	284(93.1%)	20(76.9%)
**CD4 percentage (%)**						
high	1(0.6%)	4(6.9%)	1(1.5%)	0(0%)	6(2.0%)	0(0%)
low	14(8.8%)	5(8.6%)	8(12.3%)	3(0%)	30(9.8%)	5(19.2%)
N	2(1.3%)	0(0%)	0(0%)	0(0%)	2(0.7%)	0(0%)
normal	142(89.3%)	49(84.5%)	56(86.2%)	20(100%)	267(87.5%)	21(80.8%)
**CD8 count (cell/μl)**						
low	9(5.7%)	14(24.1%)	17(26.2%)	11(50%)	51(16.7%)	13(50.0%)
N	2(1.3%)	0(0%)	0(0%)	0(0%)	2(0.7%)	0(0%)
normal	148(93.1%)	44(75.9%)	48(73.8%)	12(50.0%)	252(82.6%)	13(50.0%)
**CD8 percentage (%)**						
high	4(2.5%)	1(1.7%)	1(1.5%)	1(0%)	7(2.3%)	0(0%)
low	1(0.6%)	2(3.4%)	7(10.8%)	3(0%)	13(4.3%)	5(19.2)
N	2(1.3%)	0(0%)	0(0%)	0(0%)	2(0.7%)	0(0%)
normal	152(95.6%)	55(94.8%)	57(87.7%)	19(100%)	283(92.8%)	21(80.8%)
**CD4/CD8**						
high	1(0.6%)	6(10.3%)	7(10.8%)	3(0%)	17(5.6%)	2(7.7%)
low	9(5.7%)	1(1.7%)	3(4.6%)	1(0%)	14(4.6%)	1(3.8%)
N	2(1.3%)	0(0%)	0(0%)	0(0%)	2(0.7%)	0(0%)
normal	147(92.5%)	51(87.9%)	55(84.6%)	19(100%)	272(89.2%)	23(88.5%)
**CD9 count (cell/μl)**						
high	31(19.5%)	6(10.3%)	8(12.3%)	0(0%)	45(14.8%)	1(3.8%)
low	2(1.3%)	1(1.7%)	4(6.2%)	5(50.0%)	12(3.9%)	9(34.6%)
N	66(41.5%)	17(29.3%)	20(30.8%)	6(50.0%)	109(35.7%)	1(3.8%)
normal	60(37.7%)	34(58.6%)	33(50.8%)	12(0%)	139(45.6%)	15(57.7%)
**CD9 percentage (%)**						
high	2(1.3%)	2(3.4%)	1(1.5%)	3(0%)	8(2.6%)	5(19.2%)
low	1(0.6%)	0(0%)	1(1.5%)	4(0%)	6(2.0%)	2(7.7%)
N	66(41.5%)	17(29.3%)	20(30.8%)	6(50.0%)	109(35.7%)	1(3.8%)
normal	90(56.6%)	39(67.2%)	43(66.2%)	10(50.0%)	182(59.7%)	18(69.2%)
**CD6CD56 count (cell/μl)**						
high	4(2.5%)	1(1.7%)	4(6.2%)	0(0%)	9(3.0%)	0(0%)
low	11(6.9%)	5(8.6%)	5(7.7%)	3(0%)	24(7.9%)	9(34.6%)
N	66(41.5%)	17(29.3%)	20(30.8%)	6(50.0%)	109(35.7%)	1(3.8%)
normal	78(49.1%)	35(60.3%)	36(55.4%)	14(50.0%)	163(53.4%)	16(61.5%)
**CD6CD56 percentage (%)**						
high	2(1.3%)	0(0%)	4(6.2%)	1(0%)	7(2.3%)	3(11.5%)
low	9(5.7%)	1(1.7%)	1(1.5%)	2(0%)	13(4.3%)	0(0%)
N	66(41.5%)	17(29.3%)	20(30.8%)	6(50.0%)	109(35.7%)	1(3.8%)
normal	82(51.6%)	40(69.0%)	40(61.5%)	14(50.0%)	176(57.7%)	22(84.6%)
**IgA (g/L)**						
high	13(8.2%)	9(15.5%)	9(13.8%)	0(0%)	31(10.2%)	4(15.4%)
N	3(1.9%)	2(3.4%)	1(1.5%)	0(0%)	6(2.0%)	0(0%)
normal	143(89.9%)	47(81.0%)	55(84.6%)	23(100%)	268(87.9%)	22(84.6%)
**IgM (g/L)**						
high	4(2.5%)	1(1.7%)	1(1.5%)	0(0%)	6(2.0%)	1(3.8%)
low	11(6.9%)	10(17.2%)	16(24.6%)	10(50.0%)	47(15.4%)	3(11.5%)
N	3(1.9%)	2(3.4%)	1(1.5%)	0(0%)	6(2.0%)	0(0%)
normal	141(88.7%)	45(77.6%)	47(72.3%)	13(50%)	246(80.7%)	22(84.6%)

RBC, red blood cell; WBC, white blood cell.

**Figure 4:**
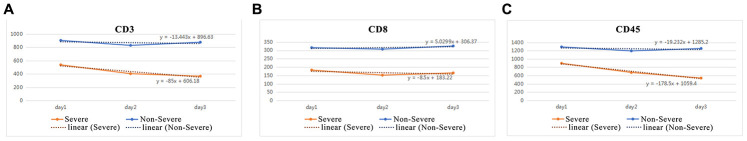
**K-values for CD3, CD8, and CD45.**

**Figure 5:**
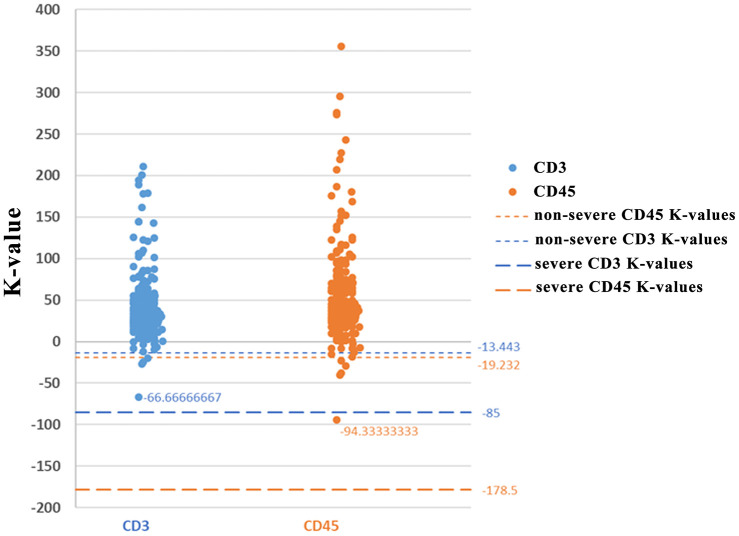
**K-value distributions of CD3 and CD45.**

Analysis of the levels of CD3 (Figure 4A), CD8 (Figure 4B), and CD45 (Figure 4C) levels in the first 3 days of analysis showed that CD3 and CD45 were more significantly decreased in severe cases compared with non-severe cases. The K-values for CD3 and CD45 in severe cases were -85 and -178.5, respectively, while the K-values for CD3 and CD45 in non-severe cases were -13.4 and -19.2, respectively. Thus, the change in K-value was most significant in the severe group, especially for CD3 (-85) and CD45 (-178.5). The analysis found that cut-off values for CD3 and CD45 were -66.7 and -94.33, respectively. A clinical test, using 80 COVID-19 patients for backward verification, revealed that the K-values for both CD3 and CD45 in non-severe cases were above the cut-off values, indicating a 100% prediction success rate for severe and non-severe cases following SARS-CoV-2 infection (Figure 5).

## DISCUSSION

The novel coronavirus SARS-CoV-2 possesses approximately 80% of the genetic sequence of SARS-CoV [7, 8]. COVID-19 is a self-limiting disease, which has also developed into a fatal disease. Respiratory droplets, close contact, aerosol, and fecal transmission are now regarded to be the primary routes of transmission [9–11]. The rapid spread of globalization has turned the disease into a global disaster. COVID-19 has caused millions of infections and hundreds of thousands of deaths. Thus, analysis of clinical case information is important in the control, prevention, and treatment of the virus.

The risk factors relevant to COVID-19 development and progression from initial infection to death included older age, inflammatory response, and immune dysfunction, consistent with previous reports [12]. The present study analyzed the clinical data of 331 COVID-19 patients. The data showed that individuals of older age (> 60 years old) were more susceptible to SARS-CoV-2 infection, consistent with previous reports [13]. The elderly are often associated with chronic diseases such as diabetes, hypertension, and coronary heart disease, and are more vulnerable. Our analysis found that the risks associated with COVID-19 increased with increasing age. The immune system weakens with increasing age; therefore, increased age is associated with a greater risk of COVID-19 infection and death, likely due to a less rigorous immune response.

Analysis of inflammatory cytokines revealed that CRP, hs-CRP, IL-1β, IL-8, IL-10, and IL-6 expression levels were increased in severe pneumonia patients, and a higher proportion of the severe pneumonia population exhibited increased levels of CRP (100%), hs-CRP (92.3%), IL-6 (61.5%), and IL-10 (84.6%) compared with non-severe patients. Although IL-10 has anti-inflammatory properties, it cannot reverse the inflammatory storm induced by COVID-19. Immunocytoanalysis found that WBCs and neutrophils were also increased, indicating an inflammatory response. Our study was consistent with previous studies which found that, in early stage COVID-19 infection, CRP levels are positively correlated to lung lesions, which could reflect disease severity [14–16]. This early increase in serum levels of pro-inflammatory cytokines was observed in SARS-CoV and Middle East Respiratory Syndrome (MERS)-CoV infection, suggesting similar cytokine storm-mediated disease severity [17, 18]. Cytokines are crucial mediators of the inflammatory response; previous reports have indicated that cytokine storm syndrome occurs with SARS-CoV-2 infection [19, 20]. This cytokine storm can initiate viral sepsis and inflammatory-induced lung injury, which can result in other complications such as pneumonitis, ARDS, shock, respiratory failure, organ failure, and death.

A number of studies have found that immune system disorders are a key factor in “cytokine storms”. Immune cell analysis found that CD3, CD45, and CD8 counts were decreased in SARS-CoV-2 infection patients. A higher proportion of severe pneumonia cases exhibited decreased CD3 (69.2%), CD45 (57.7%), and CD8 (50%) compared with non-severe patients. The first 7 days of analysis showed that severe COVID-19 patients had lower levels of CD3, CD8, and CD45. A decreased CD3 count indicates a reduction in total T cells. We also found that the lymphocyte count/percentage decreased and a higher proportion of the severe pneumonia patient population exhibited a decrease in lymphocytes (57.7%), suggesting that the immune system is damaged after SARS-CoV-2 infection. Decreased levels of CD8 indicate T-cell exhaustion, similar to hepatitis B virus (HBV) infection. It is known that HBV-specific T cells play an important role in antiviral protection and failure to control HBV is related to a dysfunctional T cell response [21]. In this manner, it is important to increase the immunity of COVID-19 patients. The present study found that CD45 counts were decreased; CD45 is necessary for T cell activation. CD45 deficiency results in B- and T-lymphocyte dysfunction in the form of severe combined immune deficiency. CD45 plays an important role in autoimmune traits and cancer as well as in infectious diseases including fungal and virus infections [22, 23]. CD45 has been shown to be decreased in density on the T cell surface during HIV infection [24]. CD45 is a transmembrane phosphatase that functions in T cell activation and signaling. Multiple CD45 isoforms are simultaneously expressed on T cells due to alternative splicing of three exons that correspond to extracellular domains A, B, and C or the exclusion of all three (CD45RO). A number of studies have reported that higher avidity CD8^+^ T cell responses are desirable in vaccine responses to pathogens and cancer, whereas the immunity persistence generated following vaccination is a factor for many pathogens. Maintaining high CD45RB expression status is indispensable for maximizing pathogen-specific immunity longevity [25–27]. This further suggests that SARS-CoV-2 can be aggressive to the immune system. Thus, a reduced level of CD45 may be a useful standard for the diagnosis of severe patients from non-severe patients.

Further analysis showed that albumin levels were decreased in COVID-19 patients, indicating hypoimmunity following SARS-CoV-2 infection [28, 29]. Dysfunction of the immune system can result in the accumulation of inflammatory factors. The inflammatory microenvironment can destroy the acid-base balance, osmotic pressure, and water distribution (decreased chlorine and increased urine protein), ultimately leading to liver and kidney damage. Multiple organ damage is a common cause of death of elderly patients infected with SARS-CoV-2 [30, 31]. The target for immune function may be the key to treatment of this disease.

How changes in immune cell number can be used to distinguish between severe and non-severe patients has important clinical significance. After analyzing the results of CD3, CD8, and CD45 levels in the first 3 days, it was found that the K-value change was the most significant in the severe group, especially for CD3 (-85) and CD45 (-178.5). The analysis found that cut-off values for CD3 and CD45 were -66.7 and -94.33, respectively. Clinical test found that the K-values for both CD3 and CD45 in non-severe case were on the up of the cut-off values, indicating a 100% prediction success rate for severe and non-severe cases following SARS-CoV-2 infection. It is likely that CD45 is more clinically meaningful to distinguish severe SARS-CoV-2 cases from non-severe cases. We believe that this standard will aid in the clinical assessment of non-severe and severe patients. Due to the limited sample size of SARS-CoV-2 cases, large sample collection will be needed in future studies for the precise prediction of severe and non-severe cases following SARS-CoV-2 infection.

## CONCLUSIONS

In conclusion, the current data suggest that immune system damage may be the pathogenic feature of COVID-19. We believe that the findings described here are essential to understand the clinical characteristics of this virus, and verify that immune system dysfunction is a cause of death following infection, particularly for elderly patients. The study also found that the K-values of CD3 and CD45 might be useful to distinguish between severe and non-severe cases. Overall, we believe that the results of the present study will help distinguish between non-severe and severe patients clinically.

## MATERIALS AND METHODS

### Patients and data collection

We conducted analysis of 311 COVID-19 patients from the Shanghai Public Health Clinical Center Affiliated to Fudan University in Shanghai, China (YJ-2020-S035-01). COVID-19 infections diagnosed from January 27, 2020 to March 9, 2020 were included. We diagnosed COVID-19 based on criteria issued by the National Health Commission of China. We divided patients into non-severe (n = 305) and severe (n = 26) cases according to the “China National Health Commission, Diagnosis and treatment of pneumonia caused by new coronavirus infection”. We obtained basic information from the electronic medical record system. Patient information such as clinical and laboratory data were extracted and used for analysis. The Ethics Committee of Shanghai Public Health Clinical Center Affiliated to Fudan University approved the study and written informed consent was obtained from all subjects.

Following the guidelines for diagnosis and treatment of novel coronavirus (2019-nCoV) infection by the National Health Commission, at the time of hospitalization, patients with either a respiratory rate > 30 breaths/min, SpO_2_ < 93% in room air, or PaO_2_/FiO_2_ ≤ 300 mmHg, were categorized as severe cases, and others were defined as non-severe cases.

### Statistical analysis

We expressed continuous data as mean ± standard deviation. We summarized categorical variables as counts and percentages in each category. We employed *t*-tests for continuous variables. We used Fisher’s exact test for categorical variables as appropriate. The Wilcoxon signed-rank test was used for K-value analysis. We included statistically significant risk factors, sex, and age in the final models. All analyses were conducted with R software. P values < 0.05 were considered statistically significant.
